# Intradural extramedullary cervical metastasis from Merkel cell carcinoma: a case report and literature review

**DOI:** 10.1186/s41016-022-00310-3

**Published:** 2022-12-02

**Authors:** Giovanni Pennisi, Andrea Talacchi, Marcello Nunzio Tirendi, Marco Giordano, Alessandro Olivi

**Affiliations:** 1 Institute of Neurosurgery, Fondazione Policlinico Universitario A. Gemelli IRCCS, Università Cattolica del Sacro Cuore, Largo Agostino Gemelli, 8, 00168 Rome, Italy; 2 Department of Neurosurgery, San Giovanni-Addolorata Hospital, Rome, Italy; 3 Department of Pathology, San Giovanni-Addolorata Hospital, Rome, Italy

**Keywords:** Spinal metastasis, Merkel cell carcinoma, Intradural spinal metastasis

## Abstract

**Background:**

Merkel cell carcinoma (MCC) is a rare aggressive primary skin carcinoma with an incidence of 44 cases per 100,000.

The natural course of MCC often results in rapid growth and early metastasis. On the other hand, the spinal cord is rarely affected and frequently features the end stage of the disease. The aim of this paper was to clarify the management of patients with spine metastasis from a skin lesion and showed a case-based update.

**Case presentation:**

A 73-year-old female was admitted to the Neurosurgical Department in December 2021 for a sudden right hemiparesis with bladder dysfunction and a history of cutaneous Merkel cell carcinoma. A magnetic resonance imaging (MRI) of the central nervous system (CNS) showed an intradural-extramedullary right-sided C6–C7 tumor with mass effect and edema of the cervical cord. The patient underwent a C6–C7 laminectomy with microsurgical total resection of the intradural extramedullary lesion. The neuropathological examination identified a metastasis from Merkel cell carcinoma. Nowadays, evidence for spinal metastasis from malignities skin cancer is generally lacking, probably because they are extremely rare. The exact time of life expectancy is controversial, and some clinicians use a cutoff of 3 months to determine whether surgical intervention should be offered, while others advocate at least 6 months of life expectancy.

**Conclusions:**

To the best of our knowledge, we showed the first case of solitary intradural extramedullary cervical spine metastasis from MCC. We recommend to consider metastasis of MCC in the differential diagnosis of spinal metastasis.

## Background

Merkel cell carcinoma (MCC) is a rare aggressive primary skin carcinoma with local invasiveness which usually involve the skin of the face and neck. The incidence of MCC is 44 cases per 100,000, and elderly patients are the most affected [1]. The common clinical features are cutaneous manifestations such as a violaceous papule, nodule, pedunculated masses, chalazion, or granulation tissue. Common treatment options include wide surgical excision and/or radiotherapy for localized disease, while chemotherapy or immunotherapies is the treatment option for metastatic disease [2].

The natural course of MCC often results in rapid growth and early metastasis, with a higher rate of local recurrence than distal metastasis [3]. The common sites described are lymph nodes, distant skin, bones, liver, and lung. If distal metastasis is uncommon, the involvement of the spinal cord is extremely rare. Furthermore, the management of these patients is often unclear and conditioned by several factors such as neurological dysfunction, intractable pain, or mechanical spine instability [4].

To the best of our knowledge, only eight papers reported an intraspinal metastasis from MCC and only two showed an intradural case of metastasis (Table 1). In this paper, we report an unusual case of solitary intradural extramedullary cervical spinal metastasis from MCC and analyze the pertinent literature review.

**Table 1:** Intraspinal metastases of Merkel cell carcinoma reported in the literature

**Authors**	**Age/gender**	**Intraspinal involvements**	**Other metastasis**	**Levels**	**Surgical treatment**	**Adjuvant therapy**	**Survival after CNS metastasis diagnosis (months)**	**Note**
Moayed et al. [5]	70/M	Epidural	LN, AOS	Sacrum	None	RT	NR	
Turgut et al. [6]	63/M	Epidural	LN, AOS	L5–S1	Laminectomy with subtotal resection	CT	2	
Vijay et al. [7]	57/F	Epidural	NR	D8, L4, S1	Laminectomy and subtotal resection	CT	1	
Ng et al. [8]	73/M	Epidural	NR	D6	Laminectomy, subtotal resection, and stabilization	None	1	
Abul-Kasim et al. [9]	65/M	Epidural, intradural	MS, BM	L3–L4	None	RT	0.5	
Madden et al. [10]	55/M	Epidural	NR	D6–D8	Laminectomy	RT	4	IC
Goodwin et al. [4]	76/M	Epidural	LN	D5	Corpectomy, tumors resection, and stabilization	RT	1.5	
Haykal T et al. [11]	49/F	Intramedullary	LN	C4–C5	None	CT, RT	NR	
Present study	73/F	Intradural extramedullary	-	C6–C7	Laminectomy and total resection	RT	5	

Abbreviations: *LN* lymph node, *AOS* associated osseous involvement, *MS* meningeal spread, *BM* brain metastasis, *IC* immunocompromised, *RT* radiotherapy, *CT* chemotherapy

## Case Presentation

A 73-year-old female was admitted to the Neurosurgical Department in December 2021 for a sudden right hemiparesis with bladder dysfunction. The patient had a history of Merkel cell carcinoma that involved the skin of the face, treated in 2016 with complete surgical resection. She underwent regular clinical and radiological follow-ups without any evidence of recurrence until January 2021, when she underwent surgical treatment of a suspicious breast lesion. The histological examination revealed a repetitive localization of Merkel cell carcinoma. Then, she was treated with adjuvant chemotherapy (monoclonal antibodies - Avelumab).

A previous radiological staging with whole-body computed tomography (CT) showed a suspicious repetitive paravertebral thoracic lesion (T12), which remained stable during clinical and radiological follow-up. The radiological features of the lesion were as follow size 0.9 x 1.1 x 0.8 cm, with moderate and homogeneous contrast-enhancing, without any mass effect on surrounding soft tissue.

Upon neurological examination, she showed sudden severe paresis of the right upper limb (Modified Research Council -MRC- 2) and mild weakness in the left upper limb (MRC 3), areflexia in the right upper limb, bilateral Babinski’s sign, hypoesthesia in lower limbs, and bladder dysfunction. She was unable to walk and needed assistance.

**Fig. 1:**
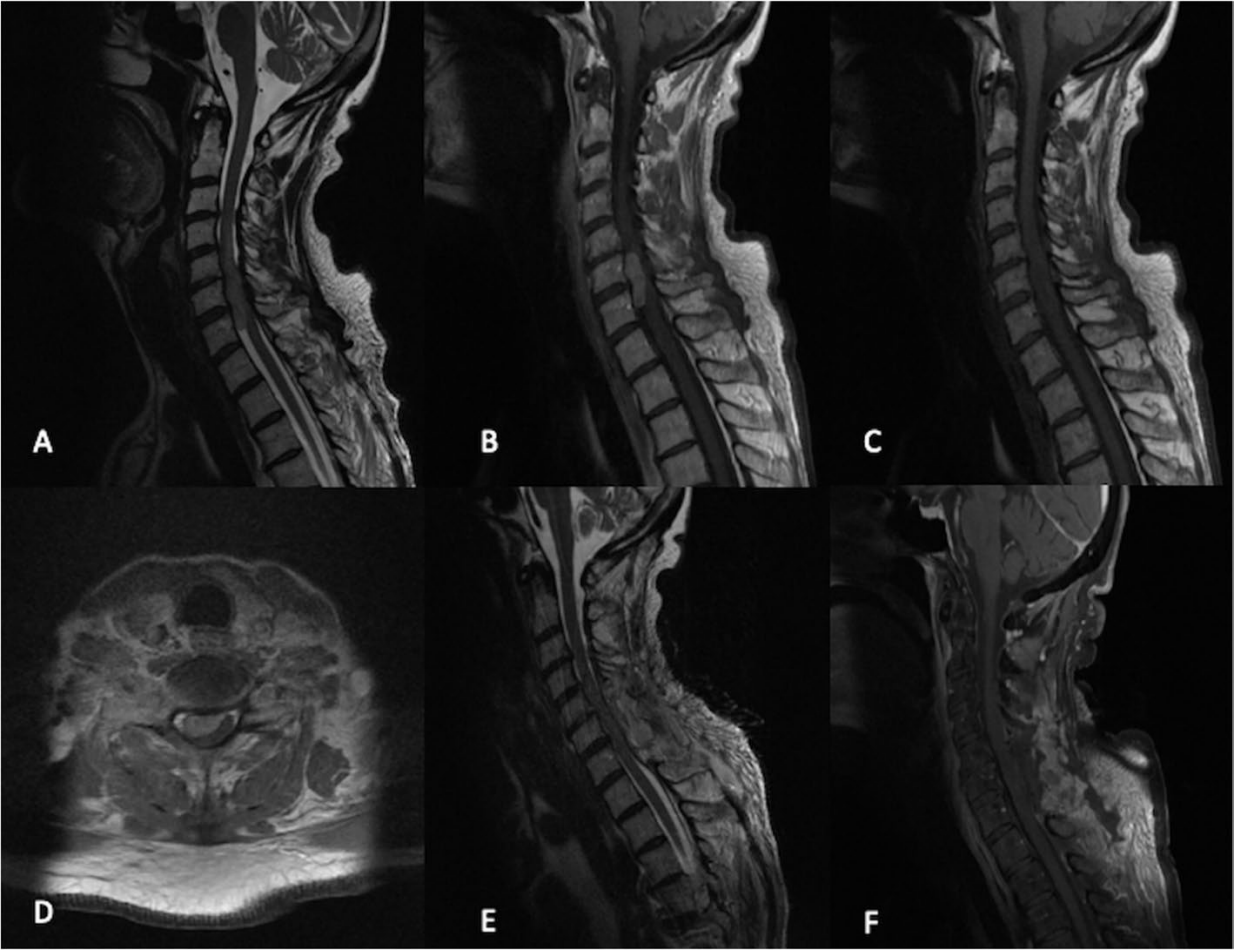
Pre-operative magnetic resonance imaging (MRI) showed an intradural-extramedullary right-sided C6-C7 lesion (**A**, **B**, **C**, **D**), with mass effect and edema of the cervical cord. T1-weighted sequences showed mild hypointense signal (**C**) and heterogenous contrast enhancement (**B**, **D**). T2-weighted sequences showed a mild hyperintense signal. Postoperative MRI (**E**, **F**) showed complete excision of the tumor

A magnetic resonance imaging (MRI) of the central nervous system (CNS) showed an intradural-extramedullary right-sided C6–C7 lesion (Fig. 1), with mass effect and edema of the cervical cord, size 2.4 × 1 × 1 cm, mild hypointense signal on T1-weighted sequences, and heterogenous contrast enhancement, furthermore a mild hyperintense signal on T2-weighted sequences. No other CNS lesion was reported neither evidence of hemorrhagic/ischemic signs.

The patient underwent a C6–C7 laminectomy with microsurgical resection of the intradural extramedullary lesion. Macroscopically, the tumor appeared compact and pale without any evidence of hemorrhagic signs. Debulking of the tumor was initially performed by an ultrasonic aspirator, and then, it was gently dissected from the medulla achieving total removal.

The postoperative neurological examination showed upper left limb paresis was slightly improved as well as sensibility in lower limbs (MRC 4). MRI confirmed a complete tumor resection without any features of postoperative complication and CSF leaks.

**Fig. 2:**
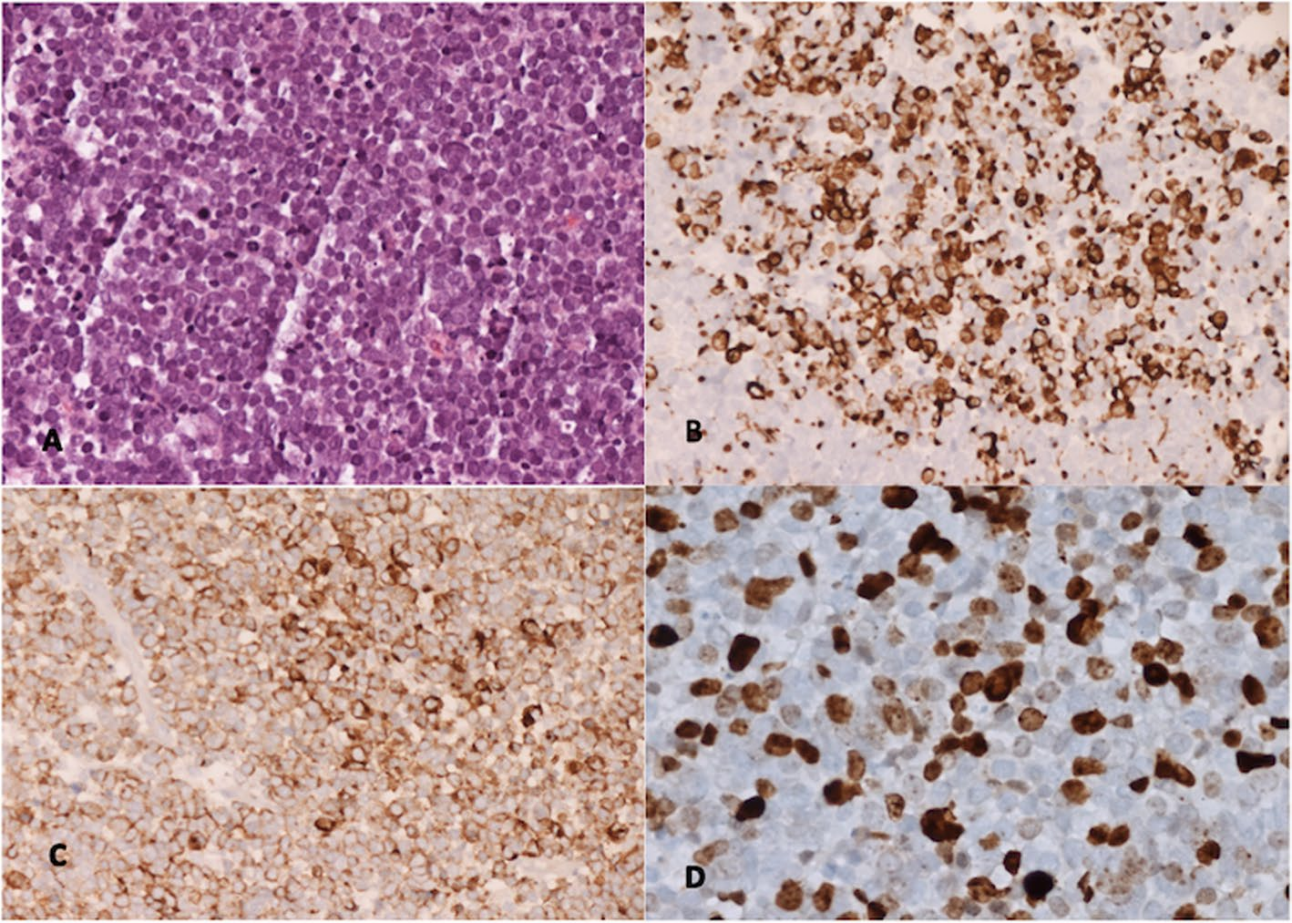
Histopathological features. **A** Monomorphic proliferations of small cells with vesicular nuclei. **B** Positive cytokeratin 20 (CK20) expression on the membrane and paranuclear site. **C** Positive expression of chromogranin on the membrane. **D** High index of proliferation with Ki67 expression

The neuropathological examination (Fig. 2) showed multiple short fragments of small cells of neuroendocrine carcinoma with a high mitotic index. There was a positive expression of focal cytokeratin 7; on the other hand, negative expression of thyroid transcription factor-1 (TTF1), gross cystic disease fluid protein 15 (GCDFDP-15), estrogen receptor (ER), progesterone receptor (PR), and HER2 (human epidermal growth factor receptor 2).

After a multidisciplinary board evaluation, the patient underwent flash radiotherapy (RT) on a previously identified lesion in T12 (8 Gy) and started the adjuvant RT treatment at the surgical site. A whole-body contrast-enhanced computed tomography (CT) scan at 3 months of follow-ups showed nodular peritoneal implants without any other localization. The patient is currently under oncological and radiological follow-up after 5 months.

## Discussion

Nowadays, surgery procedures and adjuvant therapies are the best choices for the treatment of symptomatic spinal metastases. On the other hand, clinical evidence for spinal metastasis from malignities and skin cancer is generally lacking, probably because they are extremely rare.

Moreover, the incidence of MCC has increased in the last three decades, and currently, optimal management of patients with spinal metastasis from MCC has not yet been clearly established [1].

MCC is an uncommon neuroendocrine primary skin cancer, named from the neurosecretory granules in the tumor cells that originate from a neural crest derivative of Merkel cells [12]. The first histopathological description was reported by Cyril Toker in 1972; he named the disease such as “trabecular carcinoma of the skin” [13].

The MCC is more common in Caucasians, males, and individuals greater than 65 years of age [14].

Similar to other primary skin cancers, this lesion is most commonly located in the sun-exposed areas of the skin such as the head and neck. The common clinical features are cutaneous manifestations such as a violaceous papule, nodule, pedunculated masses, chalazion, or granulation tissue. Moreover, nearly half of the patients have regional lymph node involvement at initial diagnosis and up to 3% of patients present a metastatic disease at the diagnosis [15].

The most frequent sites of distant metastasis are the lymph nodes, distant skin, lung, and bone [16].

The central nervous system (CNS) is rarely involved, despite the bone of the cranium and spine could be affected [5]. Furthermore, there are only a few cases of MCC spinal metastasis reported in the literature (Table 1). Most of the reported metastasis are localized in epidural space with concomitant involvement of adjacent bone and/or paravertebral space. In particular, when the adjacent bone and/or neural foramina appear involved, the possibility of epidural extension through these structures should be considered.

Only two authors reported an intradural location of MCC metastasis, and one of these described an intramedullary lesion in an end-stage case of MCC [11]. Abul-Kasim et al. described a patient with a leptomeningeal spread of disease and multiple brain localization that could be explained by the intradural dissemination of MCC metastasis. In our paper, we report the first case of intradural extramedullary metastasis of MCC without other localization in CNS [9].

The first step of treatment of MCC should be an excision of the primary lesion with a safe margin and prophylactic lymphadenectomy followed by irradiation to the primary site [17]. Merkel cell carcinoma is a highly radiosensitive tumor, and radiotherapy is commonly used as adjuvant therapy after surgery, while chemotherapy is reserved for systemic disease [18].

In our case, the patient was treated with wide primary surgical excision in 2016, without adjuvant treatment. In January 2021, she underwent surgical bilateral resection of the breast for repetitive lesions from MCC with successive adjuvant chemotherapy. After 10 months, she presented sudden neurological impairment due to a cervical spine intradural lesion with mass effect.

The management of patients with spinal metastasis from malignant skin cancers is controversial and often associated with poor outcomes. Several factors should be considered choosing the best treatment modality. The stage of the disease, patient’s age, overall condition, ambulatory status, and life expectancy are some to be considered.

The exact time of life expectancy is controversial, and some clinicians commonly use a cutoff of 3 months to determine whether surgical intervention should be offered, while others advocate at least 6 months of life expectancy. Decompressive surgery plus radiation is demonstrated to be the preferred treatment for patients with solitary spinal metastases with symptomatic cord compression [4].

In our case, the patient had a sudden neurological impairment, more than 3 months of life expectancy, and solitary spinal metastasis without other CNS involvement. Laminectomy and complete excision  were performed with an improvement of neurological symptoms.

When the spine stability is compromised the stabilization should be considered [4, 8]. Furthermore, when the prognosis is very poor, palliative care (CT, RT) should be the treatment of choice. Only a few articles studied the median survival of patients affected by spinal metastasis from primary skin cancer. Goodwin et al. reported a median survival of 6.3 months despite the type of treatment (surgical and/or medical), whereas the mean survival was 4.6 months, 6.9 months, and 9.1 months from diagnosis of spinal metastasis, respectively, for patients treated with surgery alone, medical treatment alone, and surgery plus adjuvant therapy [4]. A possible bias of this data was that the study comprised a preponderance of case reports and a heterogeneous cohort.

According to the literature, our patient underwent adjuvant radiotherapy treatment, and actually, she is under clinical follow-up. In summary, we reported the first case of solitary intradural extramedullary cervical spine metastasis from MCC. To improve the management of patients with spinal metastasis from skin cancers, we recommend a multidisciplinary discussion to choose the best treatment and consider the metastasis of MCC in the differential diagnosis of spinal metastasis, particularly in patients with skin lesion history. Moreover, further studies are needed to improve the management and outcome of patients with spine metastasis from MCC.

## Conclusions

To the best of our knowledge, we showed the first case of solitary intradural extramedullary cervical spine metastasis from MCC. We recommend considering metastasis of MCC in the differential diagnosis of spinal metastasis in patients with benign or malignant skin lesion history.

## Data Availability

Data sharing is not applicable to this article as no datasets were generated or analyzed during the current study.
